# Effects of sodium selenite, cysteamine, bacterially synthesized Se-NPs, and cysteamine loaded on Se-NPs on ram sperm cryopreservation

**DOI:** 10.1038/s41598-023-50221-1

**Published:** 2024-01-09

**Authors:** Tannaz Salimi, Hadi Hajarian, Hamed Karamishabankareh, Leila Soltani

**Affiliations:** Department of Animal Science, Faculty of Agricultural and Engineering Science, Razi University, Kermanshah, Iran

**Keywords:** Animal biotechnology, Nanobiotechnology, Nanoparticles

## Abstract

During the cryopreservation of sperm, the production of highly reactive oxygen species (ROS) can reduce their viability and fertility. However, the addition of antioxidants can help reduce the harmful effects of ROS. One such antioxidant is selenium, which is a co-factor of the glutathione peroxidase enzyme that is effective in scavenging ROS. Cysteamine can also take part in the structure of this enzyme. The use of nanoparticles can be less toxic to cells than their salt form. To this end, researchers synthesized Se-NPs using the streptococcus bacteria and loaded cysteamine onto the synthesized Se-NPs. The biosynthesis of Se-NPs and cysteamine loaded on Se-NPs was confirmed by UV–visible spectroscopy, X-ray diffraction (EDX), Fourier transforms infrared (FTIR) spectroscopy, and Field Emission Scanning Electron Microscope (FE-SEM). For cryopreservation, ram semen samples were diluted, and different concentrations (0, 1, 5, 25, and 125 µg/mL) of cysteamine, Se-NPs, cysteamine loaded on Se-NPs, and sodium selenite were added. An extender containing no supplement was considered as control group. After cooling the semen samples, they were frozen and stored in liquid nitrogen for evaluation. The samples were thawed and analyzed for mobility, viability, membrane and DNA integrity, and sperm abnormalities, as well as malondialdehyde level (MDA) and superoxide dismutase (SOD). The data was processed using SPSS, and a significance level of p < 0.05 was considered. The results of this experiment showed that adding 1 μg/mL of cysteamine loaded on Se-NPs to the diluent significantly increased the motility, viability, and membrane integrity and SOD of spermatozoa compared to the other treatment groups and control group, and reduced the abnormality, apoptosis, and MDA level of spermatozoa in comparison with the other treatment groups and control group (p < 0.05). In conclusion, the addition of cysteamine loaded on Se-NPs was found to improve the quality of ram sperm after cryopreservation.

## Introduction

Cryopreservation of sperm as well as other assisted reproduction technologies (artificial insemination, in vitro fertilization, and intra-cytoplasmic injection) is widely used to solve infertility problems in animals, livestock, and humans^1,2^. To increase productivity during sheep's lives, most parts of the world use reproductive techniques^3^. A variety of methods have been proposed, but artificial insemination is regarded as one of the best^4^. For long-term storage of semen and sperm, cryopreservation is an effective method. Thermal shock, ice crystal formation, oxidative stress, osmotic changes, and reorganization of proteins and lipids are caused by freezing methods^5^. An important factor that influences the quality of semen is oxidative stress. Oxidative stress affects the balance between reactive oxygen species and antioxidant defenses in the body, leading to infertility and sperm quality reduction during sperm storage. Oxidative stress is caused by free radicals^6^. Sperm cells have a small amount of cytoplasm, which means they also have a limited amount of enzymes and antioxidant systems. The sperm plasma membrane is highly susceptible to damage because of the high concentration of unsaturated fatty acids and the presence of double bonds. This can lead to a reduction in sperm concentration, motility, and morphology, and ultimately affect the fertilization process^7^.

The antioxidants in the body prevent the activity of ROS, thus protecting cells from the harmful effects of these molecules^8^. Different antioxidants are present in spermatozoa, including superoxide dismutase and glutathione oxidase^9,10^. In order to estimate the bioavailability of selenium in the body, glutathione peroxidase, which is a selenium-dependent peroxidase, must be determined^11^. The testicles have both selenium-dependent and non-selenium-dependent glutathione peroxidase enzymes^12^. In spermatogenesis, glutathione peroxidase protects sperm and cell membranes, and regulates oxidative processes by scavenging ROS^13,14^.

Selenium, a potent antioxidant, is essential to glutathione peroxidase. This material enhances sperm fertility and scavenges free radicals since it is a cofactor of antioxidant enzymes. Moreover, it is necessary for spermatogenesis and good functioning^15^. A limiting factor to the use of selenium is its bioavailability and toxicity, for example, selenium can be metabolized into hydrogen selenide, a very toxic form of selenium^16^. Nanoparticles have a relatively small size and high surface area that increases their chemical activity and makes them efficient catalysts^17^.

Selenium nanoparticles have been shown in a large number of comparative efficacy and toxicity studies to be capable of producing seleno-proteins with adequate bioavailability and minimal toxicity, such as glutathione peroxidase^18^. A chemical process for making nanoparticles requires high temperatures, an acidic pH, and undesirable chemical precursors, which may make nanoparticles unsuitable for medical applications. While biological production of selenium nanoparticles is cheaper and safer, and non-toxic biocompatible materials are used. As well as all the advantages, biological selenium nanoparticles are more stable, and since they contain organic molecules as coatings, they do not accumulate or form huge masses^19^. Enzymes, plant extracts, and microorganisms are used to create selenium nanoparticles^20,21^. In both aerobic and anaerobic conditions, microorganisms may decrease selenium oxyanions to create selenium nanoparticles. This study looks at how selenium nanoparticles affect the quality of sperm that has been frozen and thawed. Several studies have shown that supplementing semen extender with certain compounds can improve the quality and viability of cryopreserved sperm. One such compound is selenium nanoparticles (SeNPs), which have been found to increase the motility and viability of bull spermatozoa. When combined with glutathione (GSH), SeNPs have also been shown to protect bull spermatozoa from cryoinjury, resulting in improved semen motility, mitochondrial activity, plasma membrane integrity, and acrosome integrity. The co-supplementation of GSH and SeNPs has also been found to enhance the antioxidant capacity and embryonic development potential of frozen-thawed bull spermatozoa^22^. Additionally, enriching semen extender with Se-NPs has been found to improve the post-thaw sperm quality of Holstein bulls and consequently increase the in vivo fertility rate by reducing apoptosis, lipid peroxidation, and sperm damage caused by cryopreservation^23^.

Cysteamine, or 2-mercaptoethylamine, is an aminothiol produced by body cells during coenzyme A metabolism^24^. At low concentrations, cysteamine induces cysteine to enter cells and produce glutathione, which functions as an intracellular antioxidant. On the other hand, its thiol group reacts with the disulfide bonds of peptides and proteins, disrupting their function. Furthermore, cysteamine causes hydrogen peroxide production and oxidative stress, which decreases glutathione peroxidase activity at high concentrations^25^. The addition of cysteamine to sperm cryopreservation media has been found to be an effective antioxidant, improving progressive motility, total antioxidant capacity, and decreasing MDA content^26^. Another study found that adding cysteamine and ergothioneine to ram semen extender improved the post-thawing quality of cryopreserved sperm^27^.

Research in the area of bio-synthesis of nanoparticles with low toxicity range and conjugation to antioxidants has become increasingly attractive for several biomedical applications. Nano-conjugates exhibited a notable increase in antioxidant molecules activity. Many studies indicate that the combination of antioxidants with Se-NPs improves the effectiveness of antioxidant activity. The decoration of the Se-NPs surface with lichenan endowed the L-Se-NPs with superior antioxidant capability, and their free radicals scavenging ability exhibited in a dose-dependent manner. Furthermore, L-Se-NPs showed excellent selenium controlled-release performance^28^. Considering this fact, we report the bio-synthesis of Se-NPs using Staphylococcus aureus without the addition of any reducing agent and subsequent conjugation of the Se-NPs with cysteamine for the first time to enhance the antioxidant activities.

Therefore, the purpose of the present study was to determine the efficacy of different concentrations of sodium selenite, cysteamine, Se-NPs, and cysteamine loaded on Se-NPs as antioxidants on frozen-thawed ram sperm motility; total motility, viability membrane, DNA integrity, sperm abnormalities, level of malondialdehyde (MDA) and superoxide dismutase (SOD) activity.

## Materials and methods

### Chemicals

The chemicals used in this study were all obtained from Sigma-Aldrich, except otherwise that mentioned.

### Synthesis of selenium nanoparticles

One colony (*Staphylococcus aureus*) was grown overnight in nutrient broth to prepare the bacterial suspension. Next, a nutrient broth solution supplemented with 0.1M sodium selenite was inoculated with 10^7^ CFU/mL of bacterial suspension and incubated at 37 °C under static conditions for 48 h at 150 rpm. A UV–visible-spectrophotometer (T80) was used to analyze the samples. The particles were separated from the reaction mixture by centrifugation at 12,000 rpm for 10 min and then washed several times with distilled water and ethanol. Next, the sediment was poured into the plate and placed in the dark to dry, and finally, the dried sediment was collected.

### Conjugation of cysteamine with Se-NPs (Cys-Se-NPs)

To synthesize cys-SeNPs, a mixture of biosynthesized Se-NPs and cysteamine in equal volumes was prepared in ethanol at a concentration of 1 mg/mL. This mixture was stirred at room temperature for 24 h, after which the cys-SeNPs were separated by centrifugation at 7000 rpm for 20 min. The supernatant obtained from the centrifuge was analyzed using a UV–visible spectrophotometer to determine the efficiency of conjugation. An absorbance at 250 nm indicated the presence of unconjugated drug in the supernatant. To calculate the conjugation efficiency, the amount of non-conjugated drug in the supernatant was subtracted from the total amount of added drug. The standard curve equation was used to determine the conjugation efficiency of the drug.

1 $$\mathrm{Conjugation\, efficiency }= [(\mathrm{Total \,Drug }-\mathrm{ Unconjugated\, Drug}) /\mathrm{ Total \,Drug}] \times 100$$

A method has been developed to estimate drug concentrations in unknown samples by comparing them to standard samples of known concentrations. This method is reliable, simple, and reproducible. To obtain a calibration curve of cysteamine concentration in aqueous solutions, we plotted concentrations of cysteamine against absorbance (max 250 nm). The Beer-Lambert law was applied to measure the drug concentration in distilled water which ranged from 0.85 to 3.78 μg/mL. We computed line equations and plotted cysteamine's linearly reverted standard curve based on linearly reverting concentrations and absorbances. The relevant coefficient value in the standard graph was close to 0.944, which demonstrates that the medication complies with the Beer–Lambert rule within the concentration range of 0.1–0.8 μg/mL.

### Ethical approval

Experiments were done in accordance with the approved protocols, institutional guidelines for the care and use of laboratory animals and ARRIVE recommendations. Animal husbandry and handling were conducted in accordance with the guidelines of Animal Ethics Committee (Permission number: IR.RAZI.REC.1399.069) of Razi University, Kermanshah, Iran.

## Characterization

### UV–Vis spectrophotometer

UV–visible spectra were used to monitor Se ion reduction by Staphylococcus aureus via Se-NPs, using a T80 spectrophotometer for spectroscopy.

### Fourier transform infrared (FTIR)

The spectrum of Se-NPs bio-synthesized by Staphylococcus aureus bacteria and cysteamine loaded on Se-NPs were determined using a Bruker Alpha FTIR spectroscopy with KBr pellets in the range 500–4000 cm^−1^.

### FE-SEM

An FE-SEM and EDS analysis was performed to analyze the surface shape and particle elements (FE-SEM TESCAN MIRA3). Se-NP surface charges and cysteamine loaded on Se-NPs were measured using a Zeta-Potential analyzer (Zeta SZ-100).

### Animals and semen collection

Four adult Sanjabi rams (4 years old) were employed in this investigation to gather semen samples. The rams were housed under uniformly optimal nutritional conditions at Razi University, the Faculty of Agriculture, and Kermanshah. Through artificial vaginas, ejaculates were collected twice a week from rams. The ejaculates were collected and then immediately placed in a water bath at 33 °C to be inspected under a microscope. The analysis of all the semen was completed around 20 min after the semen was collected. To prevent individual variability among rams, ejaculates with a volume of 0.5–2 mL, a minimum semen concentration of 3 × 10^9^ spermatozoa/mL, forward progressive motility of more than 80%, and less than 10% aberrant sperm were chosen and combined before being utilized in the experiment. Each pool of semen was diluted with 20% egg yolk in a Tris-base extender to provide 200 × 10^6^ sperm/mL.

### Extender preparation and sperm dilution

A portion was diluted in 100 mL of distilled water with Tris (0.2975 M), citric acid (0.1053 M), fructose (0.0826 M), penicillin (100,000 IU), and streptomycin (100 mg). Then, 74 mL of this solution was combined with 6 mL of glycerol and 20 mL of egg yolk. Two mL of the diluents were spilled into 17 sterile tubes and cysteamine, Se-NPs, cysteamine loaded on Se-NPs, and sodium selenite (0, 1, 5, 25, and 125 µg/mL) were added to each tube. A supplement-free extender was used as the control group. Every treatment groups cooled slowly up to 5 °C and equilibrated for 4 h. The semen samples were then packed into 0.25 mL polyvinyl French straws manufactured by IMV, France. After the equilibration period, the straws were placed horizontally on a rack and frozen at a distance of 4 cm above liquid nitrogen (LN2) in vapor for 10 min. They were later dipped in liquid LN2. Upon thawing, the frozen straws were kept at a temperature of 37 °C for 45 s.

### MTT assay

A colorimetric test called the MTT assay is used to assess the metabolic activity of cells. Tetrazolium can be reduced by mitochondrial enzymes to an insoluble formazan^29^. Using the protocol outlined by Mohammadi and Soltani^30^, the MTT test was performed to evaluate the vitality of diluted fresh sperm. Dulbecco's phosphate-buffered saline (DPBS) was used to generate a 5 mg/mL MTT solution, which was then filtered and stored at 4 °C in the dark for a maximum of 2 weeks. Next, the sperm suspension was placed in an Eppendorf tube with 100 µL of diluted sperm and 10 µL of the MTT stock solution added. The tube was then incubated with various concentrations (0, 1, 5, 25, and 125 µg/mL) of sodium selenite, cysteamine, and Se-NPs. After a 2-h incubation period, the MTT reduction rates were measured using an ELISA reader (BioTek (Power Wave XS2)) at a wavelength of 570 nm. In order to determine the number of viable cells, a standard curve was prepared by combining varying ratios of viable and freeze-killed sperm (v/v) namely 10:0, 8:2, 6:4, 4:6, 2:8, and 0:10. The viability of sperm in each sample was then determined using the MTT test. MTT reduction rates were measured right away and after a 2-h incubation period at 37 °C. The data obtained from the experiment was used to formulate a regression equation that would describe the relationship between the MTT reduction rate and sperm viability.

### Total motility

To assess the total motility of the sperm, a phase contrast microscope was used at 400× magnification^31^. 10 µL of thawed sperm were placed on a warm slide, followed by placing it on a second slide. The percentage of total mobility was calculated by counting the number of sperm in five fields on each slide.

### Sperm viability

The viability of sperm was assessed through eosin-nigrosin staining method^32^. The staining process involved mixing 10 µL of sperm with 20 µL of stain on a warm slide and then shedding the stain with another slide. After drying, under phase-contrast microscopy (CKX41; Olympus, Tokyo, Japan), viable and non-viable spermatozoa were examined. Nonviable spermatozoa were stained purple, while viable ones were without coloration.

### Assessment of sperm membrane integrity

To test for the integrity of the sperm membrane, three drops of samples were placed in Eppendorf tubes containing 1 mL of the hypoosmotic swelling (HOS) solution. The HOS solution is composed of 62.5 mL formalin (37%), 150 mL sodium saline solution, 150 mL buffer solution, and 500 mL double-distilled water^33^. A drop of this mixture was placed on a slide and covered with a cover slip. Under phase-contrast microscopy, 200 spermatozoa were counted to determine the percentage of sperm membrane integrity at a magnification of 1000× using oil immersion.

### Sperm abnormalities

The total number of abnormalities in spermatozoa was determined by homogenizing three drops (10 µL) of semen with 1 mL of Hancock's solution^34^. After freezing and thawing, the total morphological abnormalities in sperm, such as macrocephaly, loose head, degenerated head, proximal gout, curled tail, and short tail, were examined. 15 µL of prepared sperm were placed on glass slides with coverslips to examine for complete abnormalities. The percentage of total abnormalities was determined by counting two hundred sperms under a phase-contrast microscope (1000×).

### Sperm chromatin structure assay

The denaturation of DNA was detected by staining with acridine orange. To perform this procedure, a 1% AO solution was required. The semen samples were fixed in Carnoy's solution (methanol:glacial acetic acid in a 3:1 ratio) for 3 h, washed, and air-dried. After immersing the slides in a working solution of AO (prepared daily by mixing 2.5 mL of 1% AO stock solution, 10 mL of 0.1 mol/L citric acid, and 400 L of 0.3 mol/L Na_2_HPO_4_·H_2_O), they were rinsed in water and then mounted wet. The percentage of spermatozoa with normal DNA (green fluorescence) was determined using a fluorescence microscope and an excitation wavelength of 450–490 nm. A DNA fragmentation index was calculated based on the percentage of spermatozoa with normal DNA.

### Superoxide dismutase (SOD)

The SOD activity was measured in accordance with the manufacturer's instructions, using a SOD assay kit (Teb Pazhouhan Razi). The spectrophotometer measured the SOD activity at 560 nm. The total SOD activity in each sample was calculated as units per milligram of total protein in spermatozoa (U/mg protein).

### MDA content assay

To measure lipid peroxidation, we used a kit from Teb Pazhouhan Razi (TPR) to measure MDA (malonyl dialdehyde). The samples were prepared following the instructions provided with the kit. The readings were then taken at 530 nm using an ELISA device, and the results were expressed in nmol/mg.

### Statistical analysis

The analysis was carried out using SPSS (version 16). The results of the semen analysis were expressed as means ± standard deviations. Among treatments, mean values were compared using one-way ANOVA. Data were analyzed using the Duncan test. The level of significance was set at p value < 0.05.

## Results

**Figure 1:**
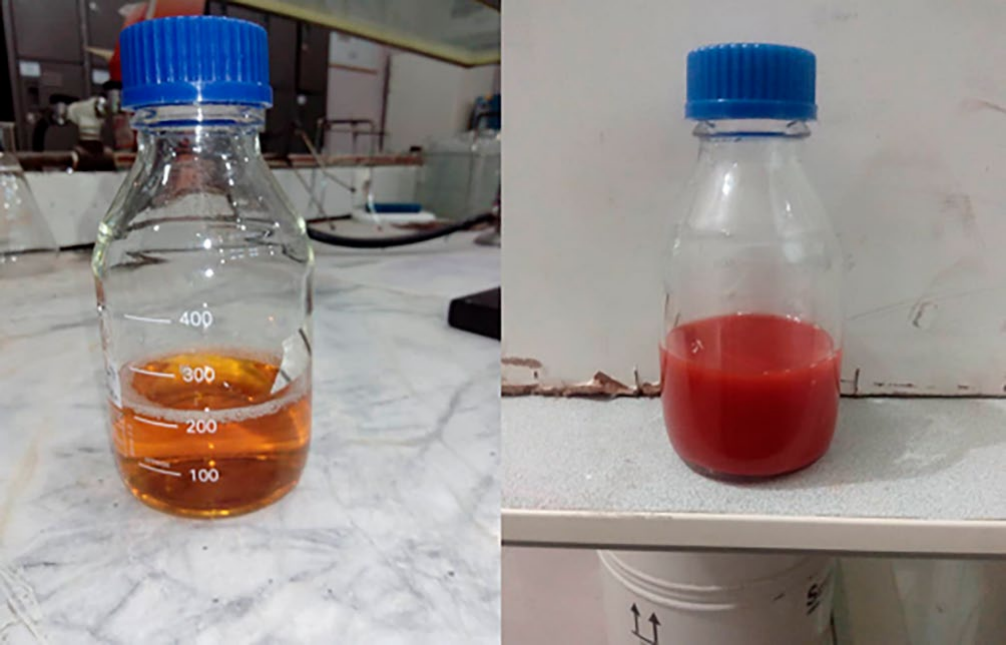
Color change of nanoselenium reaction solution.

Following the addition of sodium selenite solution, the reaction product changes color to brick in the first sign of synthesis of Se-NPs^35^. This Fig. 1 shows how the reaction solution changes color from green to brick color after being treated with sodium selenite solution for 96 h at 25 °C.

### UV–visible

**Figure 2:**
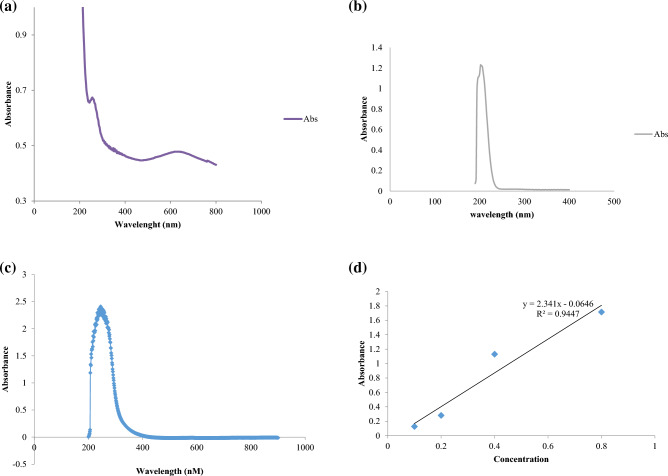
UV–Vis spectra of (**a**) bacterially synthesized Se-NPs, (**b**) cysteamine loaded on Se-NPs; (**c**) supernatant of cysteamine loaded on Se-NPs; (**d**) standard diagram of different concentrations of cysteamine.

This figure shows the absorption peak at 262 nm at the wavelength of 200–800 nm in ultraviolet–visible spectroscopy, which is related to Se-NPs, as seen in the ultraviolet–visible spectroscopy (Fig. 2a).

### Cysteamine loaded on selenium nanoparticles

The study involved analyzing the UV–Vis absorption spectra of biosynthesized Se-NPs and cysteamine loaded on Se-NPs, as shown in Fig. 2. Prior to analysis with a UV–Vis spectrophotometer, Se-NPs and cysteamine-loaded Se-NPs were mixed with double-distilled water and agitated ultrasonically for 10 min. The maximum wavelength (262 nm) of Se-NPs shifted to a shorter wavelength (202 nm), indicating that the conjugation process was successful^36^. The amount of antioxidant that loaded on Se-NPs was measured using an UV–visible spectrophotometer (Fig. 2b). The unconjugated medication was found in the supernatant after centrifugation and measured at a wavelength of 250 nm (Fig. 2c). The efficiency of the antioxidant conjugation was determined using the standard curve equation (Fig. 2d):

$$\mathrm{y }= 2.341\mathrm{x }- 0.0646$$

Based on the results, it was determined that the conjugation efficiency was approximately 32.42%.

### Field emission scanning electron microscopy

**Figure 3:**
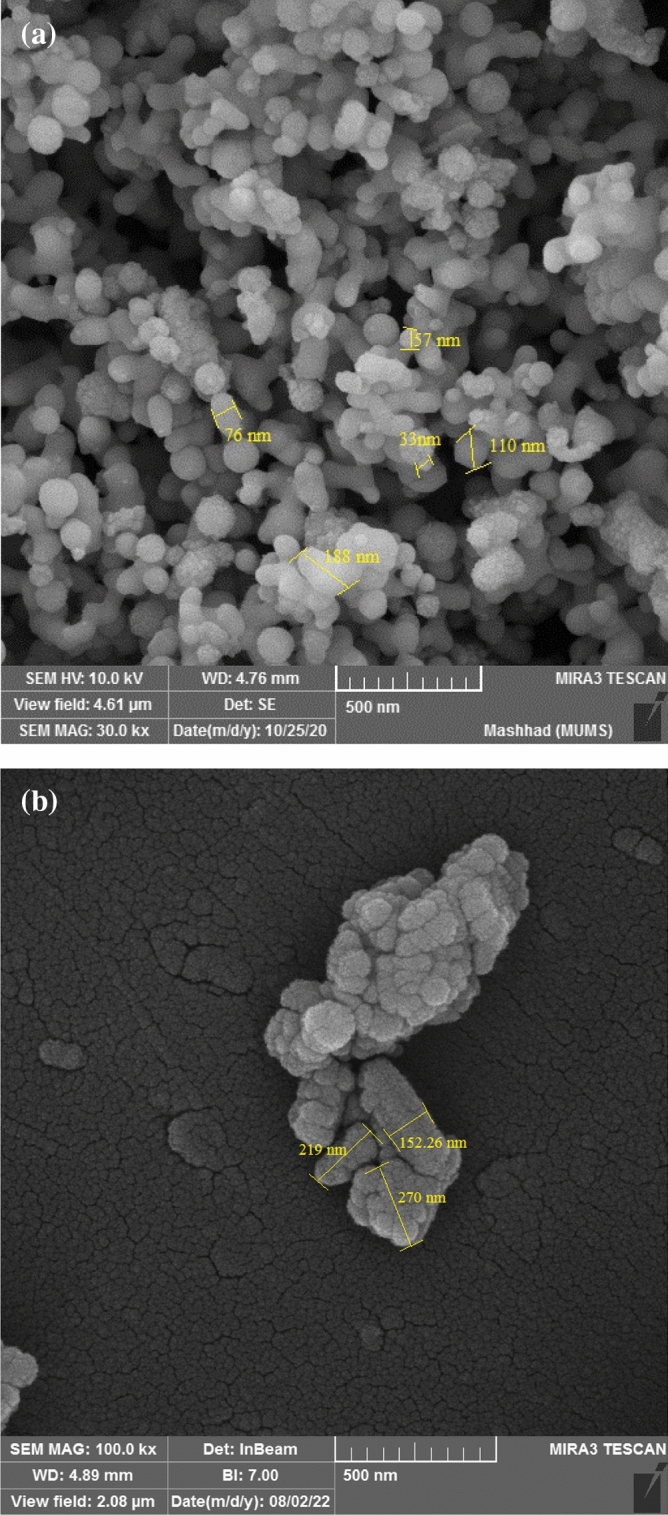
FE-SEM image of (**a**) bacterially synthesized Se-NPs, (**b**) cysteamine loaded on Se-NPs.

The scanning electron microscope images in Fig. 3 display the size and shape of Se-NPs, which were synthesized by Staphylococcus aureus bacteria (Fig. 3a) and loaded with cysteamine (Fig. 3b). The sizes of bacteria-synthesized Se-NPs and cysteamine-loaded Se-NPs were found to be 119.59 ± 7.92 nm and 250.96 ± 10.66 nm, respectively. The size of the nanoparticles was calculated using Image J 1.48v. In synthetic nanoparticles, the shape is often round and spherical, whereas the cysteamine coating changes its shape and size, which is likely due to the cysteamine coating on synthetic nanoparticles.

**Figure 4:**
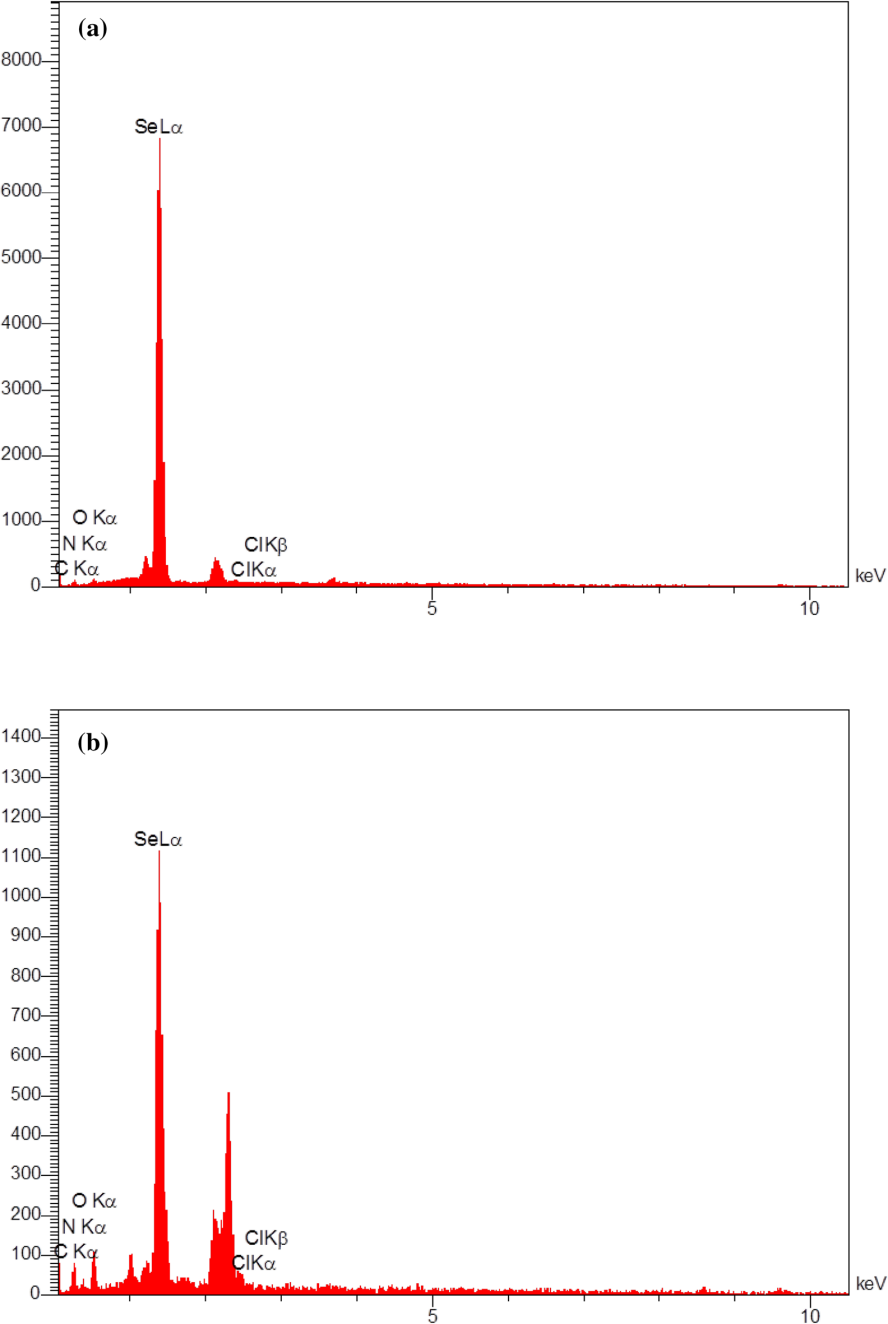
DEX analysis of (**a**) bacterially synthesized Se-NPs, (**b**) cysteamine loaded on Se-NPs.

Elemental analysis was conducted to determine the presence of selenium in the sample and to analyze the chemical composition of the produced Se-NPs, as shown in Fig. 4. This X-ray diffraction analysis determines the elements present in the sample based on the X-ray energy emitted by it. For each element, this energy has a different value. According to the elemental analysis of Se-NPs synthesized by Staphylococcus aureus bacteria (Fig. 4a), there are four main signals: one from a selenium atom (75.69%), carbon atom (12.45%), nitrogen atom (6.69%), and oxygen atom (5.19%). On the basis of the elemental analysis of cysteamine loaded on Se-NPs (Fig. 4b), four signals can be identified: a selenium atom (43.74%), a carbon atom (25.55%), a nitrogen atom (16.44%), and an oxygen atom (14.26%). For other elements, no visible peaks were observed. The change in signal number may be attributed to the cysteamine loaded on Se-NPs.

Microorganisms have the remarkable ability to synthesize metal nanoparticles. Enterococcus faecalis bacteria, in particular, have been studied for their ability to synthesize selenium nanoparticles (Se-NPs) from sodium selenite. The Se-NPs that were biosynthesized were spherical in shape and had a size range of 29–195 nm. When there were high concentrations of sodium selenite in the medium, only small amounts of selenium nanostructures were produced by bacteria. In addition, Se-NPs can be used as an anti-staphylococcal element to effectively prevent and treat *S. aureus* infections^37^.

### Zeta potential

**Figure 5:**
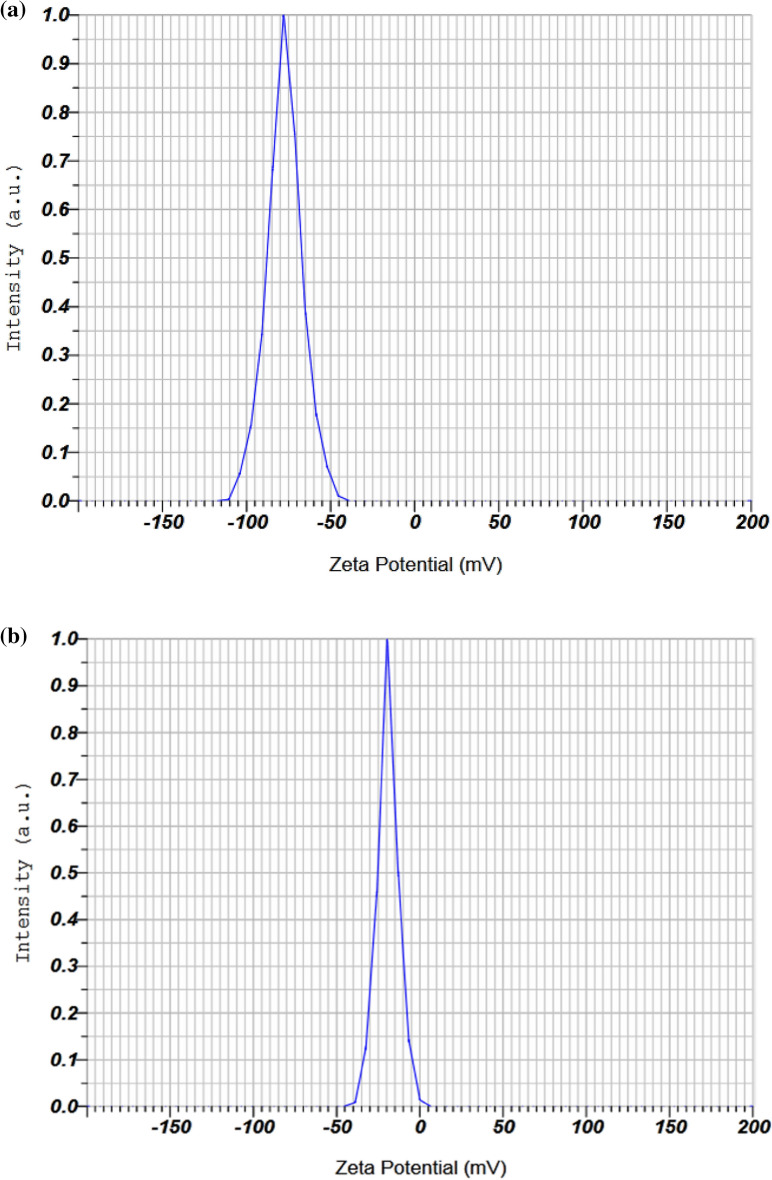
Zeta potential analysis of (**a**) bacterially synthesized Se-NPs, (**b**) cysteamine loaded on Se-NPs.

The Zeta potential was used to investigate the stabilized and surface-charged nanoparticles. Figure 5 shows a distinct peak at − 77.4 mV for Se-NPs synthesized by Staphylococcus aureus bacteria (Fig. 5a), while a peak at − 19.2 mV is observed for cysteamine loaded onto Se-NPs (Fig. 5b). The negative charges on the surface of the synthesized nanoparticles, along with other factors contributing to their stability, are primarily responsible for their stability. The cysteamine loaded on Se-NPs may be due to displacement of its negative charge compared to the Se-NPs in the present study. Overall, the results suggest that good stability is achieved through negative charges on the surface of the nanoparticles.

In a study by Borah et al.^38^, selenium nanoparticles were synthesized using Bacillus paramycoides bacteria after 72 h of culture from sodium selenite salt. The average size of the synthesized nanoparticles was 149.1 ± 29 nm, and their zeta potential was − 29.9 mV.

### Fourier transform infrared spectroscopy (FT-IR)

**Figure 6:**
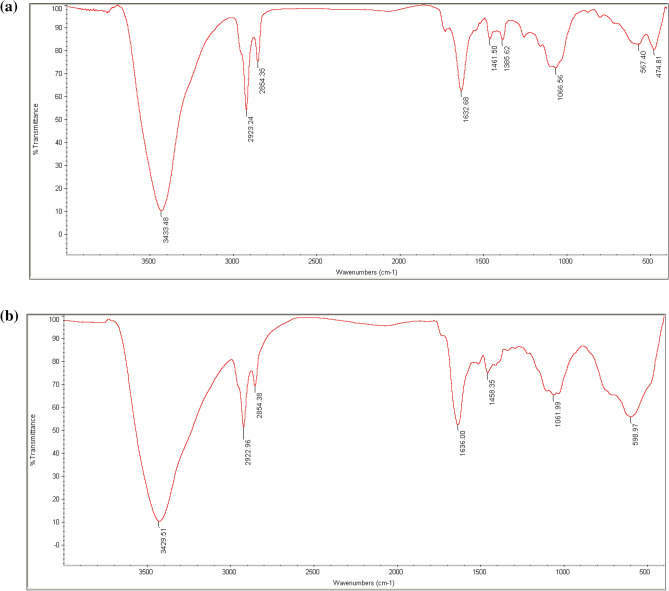
FT-IR spectra of (**a**) bacterially synthesized Se-NPs, (**b**) cysteamine loaded on Se-NPs.

In order to gain a better understanding of the process of synthesizing Se-NPs and cysteamine loaded on Se-NPs, we conducted an FT-IR analysis to identify the presence of organic substances on the surface of the synthesized nanoparticles. Figure 6a,b show the results of our analysis.

The FT-IR spectra of Se-NPs synthesized by Staphylococcus aureus bacteria (Fig. 6a) revealed several functional groups on the surface, including peaks at 474, 567, 1066, 1385, 1461, 1632, 2854, 2923, and 3433 cm^−1^, which corresponded to O–H, N–H, and O–H, primary and secondary amines, and C–H, N–H, O–H, respectively^39^.

Meanwhile, the FT-IR spectra of Cysteamine loaded on Se-NPs (Fig. 6b) exhibited different peaks at 598, 1061, 1458, 1636, 2854, 2922, and 3429, which are associated with functional groups O–H and N–H, while primary and secondary amines are C–H and C–H respectively^40^. Dobias et al.^41^ demonstrated that proteins attached to nanoparticle surfaces are important for controlling the size of selenium nanoparticles synthesized by bacteria^41^.

### MTT assay

The study aimed to test the toxicity of semen samples diluted with different compounds. The results showed that the groups that received 1 μg/mL of Se-NPs synthesized by bacteria had significantly increased sperm viability (p < 0.05) compared to the control and other treatment groups (Supplementary Table 1). The highest viability was observed in the groups that received 1 μg/mL of Se-NPs synthesized by bacteria and cysteamine loaded on Se-NPs. On the other hand, the groups that received 125 μg/mL of sodium selenite had a significantly lower sperm viability (p < 0.05) compared to the control and other treatment groups (Supplementary Table 1). The lowest viability was observed in the groups that received 125 μg/mL of sodium selenite and cysteamine loaded on Se-NPs.

### Results of the investigation of the effect of cysteamine, Se-NPs synthesized by bacteria, cysteamine loaded on Se-NPs and sodium selenite on sperm motility after frozen-thawed

The addition of 1 μg/mL of cysteamine loaded on Se-NPs to ram semen diluent significantly increased sperm total motility after the freeze-thawing process, compared to other treatment groups (Table 1). However, when sodium selenite was added to the diluent at concentrations of 25 and 125 μg/mL, the average total motility was significantly reduced (p < 0.05) after freezing and thawing, when compared to other treatment groups (Table 1).

**Table 1:** Motility and membrane integrity of sperm after the addition of sodium selenite, cysteamine, bacterially synthesized Se-NPs, and cysteamine loaded on Se-NPs as well as sodium selenite.

Treatment groups	Total motility (%)	Membrane integrity (%)
Control	56.07 ± 0.85^e^	54.80 ± 0.79^d^
1 µg/mL of cysteamine	56.23 ± 1.07f.	55.64 ± 0.97^d^
5 µg/mL of cysteamine	55.11 ± 0.84f.	55.99 ± 0.66^d^
25 µg/mL of cysteamine	67.70 ± 1.18^c^	64.72 ± 0.77^b^
125 µg/mL of cysteamine	34.11 ± 0.48^k^	31.11 ± 0.95^i^
1 µg/mL of bacterially synthesized Se-NPs	67.06 ± 1.21^b^	65.03 ± 0.86^b^
5 µg/mL of bacterially synthesized Se-NPs	61.70 ± 0.68^d^	60.44 ± 0.98^c^
25 µg/mL of bacterially synthesized Se-NPs	43.50 ± 0.81^h^	41.58 ± 1.06f.
125 µg/mL of bacterially synthesized Se-NPs	40.19 ± 0.45^j^	36.48 ± 1.15^h^
1 µg/mL of cysteamine loaded on Se-NPs	69.19 ± 0.81^a^	66.57 ± 0.83^a^
5 µg/mL of cysteamine loaded on Se-NPs	67.59 ± 0.81^c^	64.02 ± 1.58^b^
25 µg/mL of cysteamine loaded on Se-NPs	42.40 ± 1.12^i^	39.06 ± 0.46^g^
125 µg/mL of cysteamine loaded on Se-NPs	34.84 ± 0.89^k^	31.92 ± 0.72^i^
1 µg/mL of sodium selenite	63.95 ± 0.95^d^	60.45 ± 0.87^c^
5 µg/mL of sodium selenite	50.90 ± 1.44^g^	48.31 ± 1.19^e^
25 µg/mL of sodium selenite	30.09 ± 1.06^k^	31.13 ± 0.98^i^
125 µg/mL of sodium selenite	29.55 ± 1.42^L^	26.28 ± 1.35^j^

The values shown are the mean ± SD of each treatment group. ^a–l^ Means represented within the same column with various superscripts are significantly different at *p* < 0.05. N = 6 biological replicates for each treatments.

### Results of the investigation of the effect of cysteamine, Se-NPs synthesized by bacteria, cysteamine loaded on Se-NPs and sodium selenite on the sperm plasma membrane integrity after frozen-thawed

Furthermore, the addition of 1 μg/mL of cysteamine loaded on Se-NPs to the ram semen diluent improved the plasma membrane integrity of ram sperm, compared to other treatment groups (p < 0.05; Table 1). However, when 125 μg/mL of sodium selenite was added to the diluent, the integrity of the plasma membrane in spermatozoa was significantly decreased (p < 0.05) after freezing–thawing when compared to other treatment groups (Table 1).

### Results of the investigation of the effect of cysteamine, Se-NPs synthesized by bacteria, cysteamine loaded on Se-NPs and sodium selenite on the sperm viability after frozen-thawed

The addition of 1 μg/mL of cysteamine loaded on Se-NPs to the ram semen diluent significantly increased spermatozoa viability when compared to other treatment groups (Table 2). Conversely, adding sodium selenite at a concentration of 125 μg/mL to the diluent of ram semen significantly reduced sperm viability after freezing–thawing when compared to other treatment groups (p < 0.05) (Table 2).

**Table 2:** Viability and SOD activity of sperm after the addition of sodium selenite, cysteamine, bacterially synthesized Se-NPs, and cysteamine loaded on Se-NPs as well as sodium selenite.

Treatment groups	Viability (%)	SOD
Control	61.02 ± 0.87^e^	32.73 ± 0.13f.
1 µg/mL of cysteamine	58.50 ± 1.29f.	32.10 ± 0.37^g^
5 µg/mL of cysteamine	58.70 ± 1.96f.	33 ± 0.24^d^
25 µg/mL of cysteamine	68.58 ± 2^c^	35.78 ± 0.32^d^
125 µg/mL of cysteamine	35.91 ± 1.25^k^	27.50 ± 0.13^L^
1 µg/mL of bacterially synthesized Se-NPs	70.14 ± 0.46^b^	40.39 ± 0.39^a^
5 µg/mL of bacterially synthesized Se-NPs	63.94 ± 0.72^d^	36.63 ± 0.25^c^
25 µg/mL of bacterially synthesized Se-NPs	46.08 ± 0.54^h^	31.21 ± 0.19^h^
125 µg/mL of bacterially synthesized Se-NPs	41.64 ± 0.72^j^	31.03 ± 0.15^h^
1 µg/mL of cysteamine loaded on Se-NPs	72.53 ± 0.79^a^	40.53 ± 0.17^a^
5 µg/mL of cysteamine loaded on Se-NPs	68.40 ± 1.08^c^	37.89 ± 0.15^b^
25 µg/mL of cysteamine loaded on Se-NPs	44.27 ± 0.69^i^	30.44 ± 0.22^i^
125 µg/mL of cysteamine loaded on Se-NPs	36.82 ± 0.63^k^	28.77 ± 0.46^k^
1 µg/mL of sodium selenite	64.93 ± 1.06^d^	33.70 ± 0.13^e^
5 µg/mL of sodium selenite	52.28 ± 1.71^g^	31.13 ± 0.18^h^
25 µg/mL of sodium selenite	35.89 ± 0.5^k^	29.39 ± 0.72^j^
125 µg/mL of sodium selenite	32.07 ± 1.15^L^	27.48 ± 0.02^L^

The values shown are the mean ± SD of each treatment group. ^a–l^ Means represented within the same column with various superscripts are significantly different at *p* < 0.05. N = 6 biological replicates for each treatments.

### Results of the investigation of the effect of cysteamine, Se-NPs synthesized by bacteria, cysteamine loaded on Se-NPs and sodium selenite on the activity of superoxide dismutase enzyme (U/mg protein) of frozen-thawed sperm

The study showed that adding cysteamine loaded on Se-NPs, as well as Se-NPs synthesized by bacteria, to ram semen diluent significantly (p < 0.05) increased SOD activity in sperm cells when compared to other treatment groups (Table 2). On the other hand, SOD activity in sperm cells decreased significantly when 125 μg/mL of sodium selenite and cysteamine were added to the ram semen diluent, compared to the other treatment groups (p < 0.05; Table 2).

### Results of the investigation of the effect of cysteamine, Se-NPs synthesized by bacteria, cysteamine loaded on Se-NPs and sodium selenite on the production of malondialdehyde (nanomol/mg) of frozen-thawed sperm

The addition of 1 μg/mL of cysteamine loaded on Se-NPs to the ram semen diluent significantly reduced the malondialdehyde levels after the freezing–thawing process compared to other treatment groups (p < 0.05; Table3). The study showed no significant difference between 1 μg/mL of Se-NPs synthesized by bacteria and those loaded with cysteamine (p > 0.05) (Table 3). On the other hand, adding 125 μg/mL of sodium selenite to the diluent of ram semen significantly (p < 0.05) increased the level of malondialdehyde in spermatozoa cells after the freeze-thawing process compared to other treatment groups (Table 3).

**Table 3:** Total abnormality and MDA production of sperm after the addition of sodium selenite, cysteamine, bacterially synthesized Se-NPs, and cysteamine loaded on Se-NPs as well as sodium selenite.

Treatment groups	Total abnormality (%)	MDA
Control	19.88 ± 0.83^d^	10.39 ± 0.13^g^
1 µg/mL of cysteamine	19.89 ± 0.48^d^	9.97 ± 0.1f.
5 µg/mL of cysteamine	19.39 ± 0.48^d^	9.51 ± 0.23^e^
25 µg/mL of cysteamine	15.80 ± 1.13^b^	8.56 ± 0.07^cd^
125 µg/mL of cysteamine	25.81 ± 2.6f.	12.78 ± 0.02^j^
1 µg/mL of bacterially synthesized Se-NPs	15.63 ± 0.58^b^	8 ± 0.14^ab^
5 µg/mL of bacterially synthesized Se-NPs	16.40 ± 0.9^c^	8.71 ± 0.39^cd^
25 µg/mL of bacterially synthesized Se-NPs	23.16 ± 1.48^e^	10.47 ± 0.37^g^
125 µg/mL of bacterially synthesized Se-NPs	25.29 ± 2.06f.	11.78 ± 0.45^i^
1 µg/mL of cysteamine loaded on Se-NPs	13.95 ± 0.52^a^	7.96 ± 0.02^a^
5 µg/mL of cysteamine loaded on Se-NPs	16.65 ± 0.96^c^	8.35 ± 0.21^bc^
25 µg/mL of cysteamine loaded on Se-NPs	22.69 ± 1.05^d^	11.24 ± 0.03^h^
125 µg/mL of cysteamine loaded on Se-NPs	24.94 ± 1.22f.	11.73 ± 0.05^i^
1 µg/mL of sodium selenite	17.38 ± 0.97^c^	8.83 ± 0.19^d^
5 µg/mL of sodium selenite	24.61 ± 0.51^e^	11.02 ± 0.18^h^
25 µg/mL of sodium selenite	27.82 ± 1.16^e^	13.22 ± 0.15^k^
125 µg/mL of sodium selenite	28.67 ± 0.86^e^	13.94 ± 0.082^L^

The values shown are the mean ± SD of each treatment group. ^a–l^ Means represented within the same column with various superscripts are significantly different at *p* < 0.05. N = 6 biological replicates for each treatments.

### Results of the investigation of the effect of cysteamine, Se-NPs synthesized by bacteria, cysteamine loaded on Se-NPs and sodium selenite on total abnormality of frozen-thawed sperm

By adding cysteamine loaded on Se-NPs at a concentration of 1 μg/mL to the ram semen diluent, the abnormality in sperm after freeze-thawing was significantly reduced (p < 0.05) compared to other treatment groups (Table 3). However, the addition of sodium selenite at concentrations of 25 and 125 μg/mL to the ram semen diluent significantly (p < 0.05) increased the abnormalities in spermatozoa after freeze-thawing, as compared to other treatment groups (Table 3).

### Results of the investigation of the effect of cysteamine, Se-NPs synthesized by bacteria, cysteamine loaded on Se-NPs and sodium selenite on apoptosis (DNA damage) of frozen-thawed sperm

Table 4 shows that the presence of 1 μg/mL of cysteamine loaded on Se-NPs in ram semen diluent increased the percentage of spermatozoa with intact DNA compared to other treatment groups (p < 0.05). However, there was no significant difference in the percentage of spermatozoa with intact DNA between the groups treated with 1 or 5 μg/mL of cysteamine loaded on Se-NPs (p > 0.05). On the other hand, when 125 μg/mL of cysteamine loaded on Se-NPs was added to ram semen diluent, it significantly decreased the percentage of spermatozoa with intact DNA after freeze-thawing (p < 0.05). In addition, there was no significant difference (p > 0.05) in the effect of cysteamine, Se-NPs synthesized by bacteria, cysteamine loaded on Se-NPs at a concentration of 125 μg/mL, and sodium selenite salt at concentrations of 25 and 125 µg/mL on the percentage of spermatozoa with intact DNA (Table 4).

**Table 4:** DNA integrity of sperm after the addition of sodium selenite, cysteamine, bacterially synthesized Se-NPs, and cysteamine loaded on Se-NPs as well as sodium selenite.

Treatment groups	DNA integrity (%)
Control	85.63 ± 2.63^cd^
1 µg/mL of cysteamine	84.49 ± 1.92^d^
5 µg/mL of cysteamine	84.65 ± 3.79^d^
25 µg/mL of cysteamine	88.90 ± 2.44^b^
125 µg/mL of cysteamine	71.40 ± 3.4^fg^
1 µg/mL of bacterially synthesized Se-NPs	89.30 ± 0.47^ab^
5 µg/mL of bacterially synthesized Se-NPs	85.52 ± 0.90^cd^
25 µg/mL of bacterially synthesized Se-NPs	81.02 ± 1.04^e^
125 µg/mL of bacterially synthesized Se-NPs	71.09 ± 1.41^fg^
1 µg/mL of cysteamine loaded on Se-NPs	91.60 ± 0.43^a^
5 µg/mL of cysteamine loaded on Se-NPs	89.38 ± 0.69^ab^
25 µg/mL of cysteamine loaded on Se-NPs	73.31 ± 1.94f.
125 µg/mL of cysteamine loaded on Se-NPs	70.60 ± 0.88^g^
1 µg/mL of sodium selenite	87.53 ± 2.04^bc^
5µg/mL of sodium selenite	84.28 ± 2.03^d^
25µg/mL of sodium selenite	72.23 ± 1.15^fg^
125µg/mL of sodium selenite	70.70 ± 0.5^g^

The values shown are the mean ± SD of each treatment group. ^a–g^ Means represented within the same column with various superscripts are significantly different at *p* < 0.05. N = 6 biological replicates for each treatments.

## Discussion

### Motility and membrane integrity of sperm after the addition of cysteamine, bacterially synthesized Se-NPs, and cysteamine loaded on Se-NPs as well as sodium selenite

The present study observed that adding 1 µg/mL of Cysteamine loaded on Se-NPs to ram semen extenders significantly improved total motility rate and plasma membrane integrity in spermatozoa after freezing–thawing (p < 0.05). The highest motility and plasma membrane integrity were found with 5 µg/mL of Cysteamine loaded on Se-NPs and 1 µg/mL of Se-NPs synthesized by bacteria to ram semen extenders after thawing, with the exception of 1 µg/mL of Cysteamine loaded on Se-NPs. A similar finding was found in the study of Vaal Khalil et al.^23^ that investigated how Se-NPs affected the quality of bovine sperm after thawing. They found that: 0.5 and 1 µg Se-NPs were associated with a significant improvement in progressive motility and membrane integrity compared to controls. Selenium at 1 and µg/mL in diluent has also been shown to improve water buffaloes (*Bubalus bubalis*) sperm motility and membrane integrity^42^. The significant increase in sperm motility observed in the present study is probably due to selenium's effect on mitochondrial oxidative phosphorylation in sperm^43^. Oxidative stress and the peroxidation of unsaturated fatty acids in the plasma membrane are the main causes of mitochondrial dysfunction in the sperm cell. A mitochondrial function is to provide energy for the motility of sperm. Glutathione peroxidase supports these mitochondria by providing energy to the tail of the sperm, which increases the motility efficiency of the sperm. The reduction of sperm motility percentage can be explained by the disruption of the plasma membrane and sperm mitochondria in groups receiving 25 and 125 µg/mL of Cysteamine loaded on Se-NPs, Se-NPs synthesized by bacteria and sodium selenite, and 125 µg/mL of cysteamine and 5 µg/mL of sodium selenite.

### Viability and superoxide dismutase activity of sperm after the addition of cysteamine, bacterially synthesized Se-NPs, and cysteamine loaded on Se-NPs as well as sodium selenite

In the present study, the groups receiving 1 and 5 µg/mL of cysteamine loaded on Se-NPs and Se-NP synthesized using bacteria had the highest viability and SOD activity in spermatozoa cells. Kamrani et al.^44^ reported that adding Se-NPs to the extender significantly improved the viability percentage and SOD activity as compared to other treatments. These results are consistent with this research. Seleno-proteins protect the sperm membrane from damage caused by ROS and scavenge semen from ROS. A reduction in cell damage increases the percentage of live sperms and decreases the percentage of dead and damaged sperms. The results of the current study contradict those of Khoramabadi et al.^45^. In research that Khorramabadi et al.^45^ investigated the effect of adding Se-NPs in the semen extender on sperm parameters after freezing Farahani rams semen, they showed that: sperm viability after freezing–thawing was not significantly affected by the use of different levels of Se-NPs (1, 2, 4 and 8), but compared to the control group after freezing, the highest level of Se-NPs was 4%, and the lowest level was 8%. It is possible that this discrepancy is due to the fact that dosage is very important when prescribing antioxidants. A report showed that rats receiving a high level of selenium had a large number of vacuoles in their testicles, which indicates high levels of selenium are toxic, and selenium itself causes cell cycle arrest and cell death by producing ROS^46^. Antioxidants are therefore double-edged swords, whose dosage is extremely important, and whose improper use has the opposite effect^7^.

### Abnormality and malondialdehyde level of sperm after the addition of cysteamine, bacterially synthesized Se-NPs, and cysteamine loaded on Se-NPs as well as sodium selenite

In the current study, groups receiving 1 µg/mL of cysteamine loaded on Se-NPs and Se-NPs synthesized using bacteria had lower abnormalities and malondialdehyde levels in spermatozoa cells than other treatment groups, and these results are consistent with those of Lukasa et al.^47^. investigated cooling and freezing, which showed that selenium was significantly protective against lipid peroxidation (production of malondialdehyde) on sperm parameters^47^. Furthermore, Natiq et al.^48^ reported that Se-NPs treated with 1 and 2 µg/mL had lower malondialdehyde levels than the control group. Moreover, concentrations of 1 and 2 µg/mL of Se-NPs significantly improved sperm morphology and reduced abnormality when compared with controls. In order to reduce the percentage of abnormal sperm, it is necessary to enhance the integrity of sperm membranes and reduce the destructive effects of ROS and other factors that cause the oxidation of membrane lipids and eventual cell abnormalities. ROS causes cell dysfunction during membrane exchanges, and as a result, the cell is destroyed^48^.

Through the reduction of malondialdehyde, selenium improves sperm quality^49^. Malondialdehyde in semen is associated with poor sperm motility. Khalil et al.^23^ showed that adding 1 µg/mL of Se-NPs to bovine sperm extender containing Tris-egg yolk and fructose increased the total antioxidant capacity in seminal plasma and decreased malondialdehyde. According to this study, the total antioxidant capacity correlated negatively with malondialdehyde, indicating that lipid peroxidation was significantly reduced in frozen semen plasma containing Se-NPs at a concentration of 1 µg/mL^23^. It exerts its physiological actions as selenocysteine, which is derived from selenoprotein's primary structure^50^. In the sperm mitochondrial capsule, seleno-proteins are mostly phospholipid hydroperoxidase and glutathione peroxidase, which act both as enzymes as well as structural elements, primarily as active enzymes protecting the sperm against lipid peroxidation (producing malondialdehyde) and secondarily as an inactive enzyme protein that has a structural role in the formation of the mitochondrial capsule^51^. It has been reported that the activity of the seminal glutathione peroxidase enzyme relates positively to the percentage of sperm with healthy membranes, as well as to the percentage of sperm with normal morphology^52^. Ultimately, glutathione peroxidase protects sperm membranes from lipid peroxidation, resulting in an increase in sperm function.

In the present study, adding 1 and 5 µg/mL of cysteamine to the diluent did not significantly differ from the control treatment in terms of sperm abnormality, and this is in accordance with Abdollahi et al.'s^53^ findings. According to the study by Abdollahi et al.^53^, cysteamine antioxidants have no effect on sperm morphology after freezing–thawing. Furthermore, adding 1 and 5 µg/mL of cysteamine to the semen extender significantly (p < 0.05) reduced malondialdehyde levels in sperm compared to the control group, which is consistent with Abdollahi et al.^53^. The researchers found that when cysteamine was used in sperm freezing extenders, the lowest levels of malondialdehyde production, an indicator of membrane lipid peroxidation, were found in groups receiving 2 mM cysteamine, which is the difference between other treatment groups.

This study found that adding 125 µg/mL of cysteamine significantly increased malondialdehyde levels in the sperm (p < 0.05) compared to the control group, in agreement with Abdollahi et al.^53^. According to their findings, 8 mM of cysteamine caused the highest level of malondialdehyde production, indicating the negative effects of this antioxidant. A high concentration of cysteamine can increase membrane fluidity unfavorably, which makes sperm more susceptible to lipid peroxidation and causes adverse effects^54^.

### DNA integrity of sperm after the addition of cysteamine, bacterially synthesized Se-NPs, and cysteamine loaded on Se-NPs as well as sodium selenite

According to the findings of this study, the groups receiving 1 µg/mL cysteamine loaded on Se-NPs and Se-NPs synthesized by bacteria, along with 5 µg/mL Se-NPs synthesized by bacteria, experienced the lowest rates of spermatozoa cell apoptosis, which is consistent with other studies. According to Vaal Khalil et al.^23^, treated sperm cells with 1 µg/mL of Se-NPs showed a lower percentage of early apoptosis, necrotic, and apoptotic sperm cells than controls^23^. The study by Hozyen et al.^55^ showed that 0.5 µg/mL of selenium provided the greatest protection against sperm DNA damage^55^.

Overall, the present study found that adding 1 µg/mL cysteamine loaded on Se-NPs improved sperm characteristics, such as motility, viability, DNA integrity, abnormality, and SOD activity, as well as reducing malondialdehyde levels. The glutathione peroxidase enzyme is dependent on selenium as an antioxidant^56^. Seleno-proteins scavenge ROS from semen and protect the sperm membrane. By reducing cell damage, the percentage of viable sperm increases and the percentage of dead and damaged sperm decreases^57^. Dorostkar et al.^42^ concluded in a study that nanoparticles of selenium at very low concentrations significantly improved sperm quality compared with sodium selenite^42^. Due to their smaller size, selenium nanoparticles can remove a larger percentage of free radicals by improving access and activity in antioxidant systems^18^. As a glutathione synthesizer, cysteamine can play a significant role in reducing ROS as a glutathione supplement. Cysteine is an amino acid that contains sulfur (thiol group), proven to have antioxidant properties in the sperm of cobs, roosters, and rams^58,59^. Cysteine scavenges free radicals from sperm cells in order to reduce cell damage. In this regard, in research, Uysal and Bucak^60^ reported that adding cysteine to ram sperm diluent increased their viability rate^60^. According to Partyka et al.^61^, cysteine added to rooster sperm diluent improved total and progressive motility and viability^61^. In addition, Funahashi et al.^62^ observed that adding cysteine to the diluent improved pig sperm viability^62^. High concentrations of cysteamine produce hydrogen peroxide, which increases oxidative stress and decreases glutathione peroxidase activity. Low concentrations of cysteamine cause cysteine to enter the cell, which then produces glutathione as an intracellular antioxidant^63^. Thus, it is reasonable to conclude that 1 µg/mL cysteamine loaded on Se-NPs enhances sperm parameters such as motility, viability, DNA and plasma membrane integrity, abnormality, and SOD activity and reduced malondialdehyde levels than other treatments. The research findings indicate that out of the four levels of cysteamine, Se-NPs, cysteamine loaded on Se-NPs, and sodium selenite, the lowest level of mobility, viability, plasma membrane integrity, and SOD activity was observed at a concentration of 125 µg/mL of sodium selenite salt. Additionally, this concentration resulted in the highest level of MDA, which caused abnormality and cell death.

### Supplementary Information

Supplementary Information.

## Data Availability

The data that support the findings of this study are available from the corresponding upon reasonable request.

## References

[1] Kaneko T, Whittingham DG, Overstreet JW, Yanagimachi R (2003). Tolerance of the mouse sperm nuclei to freeze-drying depends on their disulfide status. Biol. Reprod..

[2] Barbas JP, Mascarenhas RD (2009). Cryopreservation of domestic animal sperm cells. Cell Tissue Bank.

[3] Sookhthezary A, Vojgani M, Niassari-Naslaji A (2006). Evaluation of using melatonin implant in rams in non-breeding season on improvement of reproductive performance in the ewes. J. Vet. Res..

[4] Adams NR (1990). Permanent infertility in ewes exposed to plant oestrogens. Aust. Vet. J..

[5] Vishwanath R, Shannon P (2000). Storage of bovine semen in liquid and frozen state. Anim. Reprod. Sci..

[6] Bucak MN, Sarıözkan S, Tuncer PB, Ulutaş PA, Akçadağ Hİ (2009). Effect of antioxidants on microscopic semen parameters, lipid peroxidation and antioxidant activities in Angora goat semen following cryopreservation. Small Rumin. Res.

[7] Agarwal A, Nallella KP, Allamaneni SS, Said TM (2004). Role of antioxidants in treatment of male infertility: An overview of the literature. Reprod. Biomed. Online.

[8] Lewis SE, Sterling ESL, Young IS, Thompson W (1997). Comparison of individual antioxidants of sperm and seminal plasma in fertile and infertile men. Fertil. Steril..

[9] Barati E, Nikzad H, Karimian M (2020). Oxidative stress and male infertility: Current knowledge of pathophysiology and role of antioxidant therapy in disease management. Cell. Mol. Life Sci.

[10] Garrido N, Meseguer M, Simon C, Pellicer A, Remohi J (2004). Pro-oxidative and anti-oxidative imbalance in human semen and its relation with male fertility. Asian J. Androl..

[11] Pelyhe C, Mézes M (2013). Myths and facts about the effects of nano selenium in farm animals–mini-review. Eur. Chem. Bull..

[12] Radostits OM, Gay C, Hinchcliff KW, Constable PD (2006). Veterinary Medicine E-Book: A textbook of the diseases of cattle, horses, sheep, pigs and goats. Health Sci..

[13] Crisol L (2012). Glutathione peroxidase activity in seminal plasma and its relationship to classical sperm parameters and in vitro fertilization-intracytoplasmic sperm injection outcome. Fertil. Steril..

[14] Tórtora-Pérez JL (2010). The importance of selenium and the effects of its deficiency in animal health. Small Rumin. Res..

[15] Burk RF, Hill KE, Motley AK (2003). Selenoprotein metabolism and function: Evidence for more than one function for selenoprotein P. J. Nutr..

[16] Tarze A (2007). Extracellular production of hydrogen selenide accounts for thiol-assisted toxicity of selenite against Saccharomyces cerevisiae. J. Biol. Chem..

[17] Adedara IA, Abiola MA, Adegbosin AN, Odunewu AA, Farombi EO (2019). Impact of binary waterborne mixtures of nickel and zinc on hypothalamic-pituitary-testicular axis in rats. Chemosphere.

[18] Safa S, Moghaddam G, Jozani RJ, Kia HD, Janmohammadi H (2016). Effect of vitamin E and selenium nanoparticles on post-thaw variables and oxidative status of rooster semen. Anim. Reprod. Sci..

[19] Wadhwani SA, Shedbalkar UU, Singh R, Chopade BA (2016). Biogenic selenium nanoparticles: Current status and future prospects. Appl. Microbiol. Biotechnol..

[20] Mishra RR (2011). Reduction of selenite to red elemental selenium by moderately halotolerant *Bacillus megaterium* strains isolated from *Bhitarkanika mangrove* soil and characterization of reduced product. Chemosphere.

[21] Ramamurthy CH (2013). Green synthesis and characterization of selenium nanoparticles and its augmented cytotoxicity with doxorubicin on cancer cells. Bioprocess Biosyst. Eng..

[22] Li S (2023). Glutathione and selenium nanoparticles have a synergistic protective effect during cryopreservation of bull semen. Front. Vet. Sci..

[23] Khalil WA, El-Harairy MA, Zeidan AE, Hassan MA (2019). Impact of selenium nano-particles in semen extender on bull sperm quality after cryopreservation. Theriogenology.

[24] Atallah C, Charcosset C, Greige-Gerges H (2020). Challenges for cysteamine stabilization, quantification, and biological effects improvement. J. Pharm. Anal..

[25] Almasi S, Rezvanjoo B, Shirazibeheshtiha SH, Namvaran AbbasAbad A, Khosravi M (2014). Protective effect of coenzyme Q10 and vitamin c on cysteamine induced lipid peroxidation. J. Vet. Clin. Res..

[26] Behmanesh MA, Janati S, Ghorbanzadeh B, Baniasadian A, Poormoosavi SM (2023). Cysteamine mitigates the deleterious impact of cryopreservation on sperm parameters. NU Mon..

[27] Najafi A (2014). Different concentrations of cysteamine and ergothioneine improve microscopic and oxidative parameters in ram semen frozen with a soybean lecithin extender. Cryobiology.

[28] Yang Z (2023). Structure, stability, antioxidant activity, and controlled-release of selenium nanoparticles decorated with lichenan from *Usnea longissima*. Carbohydr. Polym..

[29] Ghasemi M, Turnbull T, Sebastian S, Kempson I (2021). The MTT assay: Utility, limitations, pitfalls, and interpretation in bulk and single-cell analysis. Int. J. Mol. Sci..

[30] Mohammadi T, Soltani L (2021). Effects of hydroethanolic extracts of *Terminalia chebula* and *Thymbra spicata* on ram fresh semen under normal and oxidative stress conditions. Vet. Med. Sci..

[31] Zhao LL (2017). Reproductive effects of cadmium on sperm function and early embryonic development in vitro. PLoS One.

[32] Moradi M, Hajarian H, Karamishabankareh H, Soltani L, Soleymani B (2022). Pre-treatment of ram semen extender with magnetic nanoparticles on freeze-thawed spermatozoa. Vet. Med. Sci..

[33] Schäfer S, Holzmann A (2000). The use of transmigration and Spermac™ stain to evaluate epididymal cat spermatozoa. Anim. Reprod. Sci..

[34] Zhu Z (2015). Vitamin E analogue improves rabbit sperm quality during the process of cryopreservation through its antioxidative action. PLoS One.

[35] Wang H (2020). Prooxidation and cytotoxicity of selenium nanoparticles at nonlethal level in Sprague–Dawley rats and buffalo rat liver cells. Oxid. Med. Cell. Longev..

[36] Kumar CG, Poornachandra Y (2015). Biodirected synthesis of Miconazole-conjugated bacterial silver nanoparticles and their application as antifungal agents and drug delivery vehicles. Colloids Surf. B.

[37] Shoeibi S, Mashreghi M (2017). Biosynthesis of selenium nanoparticles using *Enterococcus faecalis* and evaluation of their antibacterial activities. J. Trace Elem. Med. Biol..

[38] Borah SN (2021). Selenite bioreduction and biosynthesis of selenium nanoparticles by *Bacillus paramycoides* SP3 isolated from coal mine overburden leachate. Environ. Pollut..

[39] Soltani L, Darbemamieh M (2021). Anti-proliferative, apoptotic potential of synthesized selenium nanoparticles against breast cancer cell line (MCF7). Nucleosides Nucleotides Nucleic Acids.

[40] Jafarirad S, Rasoulpour I, Divband B, Hammami Torghabe I, Kosari-Nasab M (2018). Innovative biocapped CuO nano-photocatalysts: A rapid and green method for photocatalytic degradation of 4-nitrophenol. Mater. Res. Innov..

[41] Dobias J, Suvorova EI, Bernier-Latmani R (2011). Role of proteins in controlling selenium nanoparticle size. Nanotechnology.

[42] Dorostkar K, Alavi-Shoushtari SM, Mokarizadeh A (2012). Effects of in vitro selenium addition to the semen extender on the spermatozoa characteristics before and after freezing in water buffaloes (*Bubalus bubalis*). Vet. Res. Forum..

[43] Nasri S, Amidi F, Rezaeian Movahed Z (2014). Effect of selenium on the motility, morphology and viability of sperm cells after freezing and thawing procedure. J. Inflamm. Res..

[44] Kamrani N, Karimi A, Sheikhlou MR (2021). Effects of extrinsic selenium nanoparticles on the qualitative parameters of frozen-thawed sperm of broiler breeder roosters under oxidative stress conditions. Res. Anim. Prod..

[45] Khoram Abadi F, Khodaei Motlagh M, Moradi MH (2017). Effect of in vitro selenium nanoparticles addition to the semen extender on the spermatozoa parameters after freezing in Farahani ram. J. Anim. Res..

[46] Kaushal N, Bansal MP (2007). Dietary selenium variation-induced oxidative stress modulates CDC2/cyclin B1 expression and apoptosis of germ cells in mice testis. J. Nutr. Biochem..

[47] Lukusa K, Hassen A, Lehloenya KC (2021). Dietary selenium supplementation, clarified egg yolk extender and slow cooling improve cryopreserved sperm characteristics of Saanen buck. Asian Pac. J. Reprod..

[48] Nateq S, Moghaddam G, Alijani S, Behnam M (2020). The effects of different levels of Nano selenium on the quality of frozen-thawed sperm in ram. J. Appl. Anim. Res..

[49] Huang YL, Tseng WC, Cheng SY, Lin TH (2000). Trace elements and lipid peroxidation in human seminal plasma. Biol. Trace Elem. Res..

[50] Carlson BA (2009). Selenoproteins regulate macrophage invasiveness and extracellular matrix-related gene expression. BMC Immunol..

[51] Ursini F (1999). Dual function of the selenoprotein PHGPx during sperm maturation. Science.

[52] Eidi M (2007). Effect of seminal plasma selenium concentration on semen parameters. Med. Sci. J. Islamic Azad Univ. Tehran Med. Branch.

[53] Abdollahi Z, Masoudi R, Dadashpour Davachi N (2020). Effect of cysteamine antioxidant on cellular parameters and frozen sperm quality in ram. Vet. Res. Bio Prod..

[54] Sikka SC (1996). Oxidative stress and role of antioxidants in normal and abnormal sperm function. Front. Biosci..

[55] Hozyen HF, El-Shamy AA, Farghali AA (2019). In vitro supplementation of nano selenium minimizes freeze-thaw induced damage to ram spermatozoa. Int. J. Vet. Sci..

[56] Brigelius-Flohé R, Flohé L (2020). Regulatory phenomena in the glutathione peroxidase superfamily. Antioxid. Redox Signal..

[57] Mohammadi S (2013). Effects of Vitamin-E treatment on CatSper genes expression and sperm quality in the testis of the aging mouse. Iran. J. Reprod. Med..

[58] Sarıözkan S, Bucak MN, Tuncer PB, Ulutaş PA, Bilgen A (2009). The influence of cysteine and taurine on microscopic–oxidative stress parameters and fertilizing ability of bull semen following cryopreservation. Cryobiology.

[59] Bucak MN (2007). The influence of trehalose, taurine, cysteamine and hyaluronan on ram semen: Microscopic and oxidative stress parameters after freeze–thawing process. Theriogenology.

[60] Uysal O, Bucak MN (2007). Effects of oxidized glutathione, bovine serum albumin, cysteine and lycopene on the quality of frozen-thawed ram semen. Acta Vet. Brno.

[61] Partyka A, Niżański W, Bajzert J, Łukaszewicz E, Ochota M (2013). The effect of cysteine and superoxide dismutase on the quality of post-thawed chicken sperm. Cryobiology.

[62] Funahashi H, Sano T (2005). Select antioxidants improve the function of extended boar semen stored at 10 C. Theriogenology.

[63] Besouw M, Masereeuw R, van den Heuvel L, Levtchenko E (2013). Cysteamine : An old drug with new potential. Drug Discov. Today.

