# Supplementary material for: Effects of sodium selenite, cysteamine, bacterially synthesized Se-NPs, and cysteamine loaded on Se-NPs on ram sperm cryopreservation
Source: Sci Rep. 2024 Jan 9;14:852. doi: 10.1038/s41598-023-50221-1 (PMC10774310; doi:10.1038/s41598-023-50221-1)
Supplement: Supplementary file 1 — Supplementary Information. [file 41598_2023_50221_MOESM1_ESM.docx]

Supplementary Table [1](https://www.nature.com/articles/s41598-023-47092-x#MOESM1): Analysis of viability of ram fresh sperm using toxicity assay (MTT) after adding different concentrations of sodium selenite, cysteamine, bacterially synthesized Se-NPs, and cysteamine loaded on Se-NPs to ram semen diluent

| Treatment groups | Number of cells |
| --- | --- |
| Control | 424028.5±4123.48fg |
| 1µg/mL of cysteamine | 427021.9±1897.4ef |
| 5µg/mL of cysteamine | 427799±5737.15ef |
| 25µg/mL of cysteamine | 443939.5±4303.53bc |
| 125µg/mL of cysteamine | 401446.7±2954.44hi |
| 1µg/mL of bacterially synthesized Se-NPs | 471132.4±6519.44a |
| 5µg/mL of bacterially synthesized Se-NPs | 419555±6739.39fg |
| 25µg/mL of bacterially synthesized Se-NPs | 41386.5±6554.55g |
| 125µg/mL of bacterially synthesized Se-NPs | 398418.8±6739.73hi |
| 1µg/mL of cysteamine loaded on Se-NPs | 449464.7±5299.17b |
| 5µg/mL of cysteamine loaded on Se-NPs | 434938 ±8826.1de |
| 25µg/mL of cysteamine loaded on Se-NPs | 403576.7±2174.19h |
| 125µg/mL of cysteamine loaded on Se-NPs | 394187.5±1838.57ij |
| 1µg/mL of sodium selenite | 440026.4±1186.39cd |
| 5µg/mL of sodium selenite | 419584.1±6672.78fg |
| 25µg/mL of sodium selenite | 396746±1615.18hi |
| 125µg/mL of sodium selenite | 388429±395.68j |
